# Characterization of glycosylphosphatidylinositol biosynthesis defects by clinical features, flow cytometry, and automated image analysis

**DOI:** 10.1186/s13073-017-0510-5

**Published:** 2018-01-09

**Authors:** Alexej Knaus, Jean Tori Pantel, Manuela Pendziwiat, Nurulhuda Hajjir, Max Zhao, Tzung-Chien Hsieh, Max Schubach, Yaron Gurovich, Nicole Fleischer, Marten Jäger, Sebastian Köhler, Hiltrud Muhle, Christian Korff, Rikke S. Møller, Allan Bayat, Patrick Calvas, Nicolas Chassaing, Hannah Warren, Steven Skinner, Raymond Louie, Christina Evers, Marc Bohn, Hans-Jürgen Christen, Myrthe van den Born, Ewa Obersztyn, Agnieszka Charzewska, Milda Endziniene, Fanny Kortüm, Natasha Brown, Peter N. Robinson, Helenius J. Schelhaas, Yvonne Weber, Ingo Helbig, Stefan Mundlos, Denise Horn, Peter M. Krawitz

**Affiliations:** 1 Institut für Medizinische Genetik und Humangenetik, Charité Universitätsmedizin Berlin, 13353 Berlin, Germany; 2 Max Planck Institute for Molecular Genetics, 14195 Berlin, Germany; 3 Berlin-Brandenburg School for Regenerative Therapies, Charité Universitätsmedizin Berlin, 13353 Berlin, Germany; 4 Institute for Genomic Statistics and Bioinformatics, University Hospital Bonn, Rheinische Friedrich-Wilhelms-Universität Bonn, 53127 Bonn, Germany; 5 Department of Neuropediatrics, University Medical Center Schleswig Holstein, 24105 Kiel, Germany; 6 Berlin Institute of Health (BIH), 10178 Berlin, Germany; 7 FDNA Inc., Boston, MA USA; 8 Unité de Neuropédiatrie, Université de Genève, CH-1211 Genève, Switzerland; 9 Danish Epilepsy Centre, DK-4293 Dianalund, Denmark; 10 Institute for Regional Health Services Research, University of Southern Denmark, DK-5000 Odense, Denmark; 11 Department of Pediatrics, University Hospital of Hvidovre, 2650 Hvicovre, Denmark; 12 Service de Génétique Médicale, Hôpital Purpan, CHU, 31059 Toulouse, France; 13 Greenwood Genetic Center, SC29646 Greenwood, USA; 14 Genetische Poliklinik, Universitätsklinik Heidelberg, 69120 Heidelberg, Germany; 15 St. Bernward Krankenhaus, 31134 Hildesheim, Germany; 16 Kinderkrankenhaus auf der Bult, Hannoversche Kinderheilanstalt, 30173 Hannover, Germany; 17 Department for Clinical Genetics, Erasmus MC, 3000 Rotterdam, Netherlands; 18 Institute of Mother and Child Department of Molecular Genetics, 01-211 Warsaw, Poland; 19 Neurology Department, Lithuanian University of Health Sciences, 50009 Kaunas, Lithuania; 20 Institute of Human Genetics, University Medical Center Hamburg-Eppendorf, 20246 Hamburg, Germany; 21 Victorian Clinical Genetics Services, Royal Children’s Hospital, MCRI, Parkville, Australia; 22 Department of Clinical Genetics, Austin Health, Heidelberg, Australia; 23 The Jackson Laboratory for Genomic Medicine, 06032 Farmington, USA; 24 Departement of Neurology, Academic Center for Epileptology, 5590 Heeze, The Netherlands; 25 Department of Neurology and Epileptology and Hertie Institute for Clinical Brain Research, University Tübingen, 72076 Tübingen, Germany; 26 Pediatric Neurology, Children’s Hospital of Philadelphia, 3401 Philadelphia, USA

**Keywords:** GPI, Anchor biosynthesis defects, Automated image analysis, Gene, Prediction

## Abstract

**Background:**

Glycosylphosphatidylinositol biosynthesis defects (GPIBDs) cause a group of phenotypically overlapping recessive syndromes with intellectual disability, for which pathogenic mutations have been described in 16 genes of the corresponding molecular pathway. An elevated serum activity of alkaline phosphatase (AP), a GPI-linked enzyme, has been used to assign GPIBDs to the phenotypic series of hyperphosphatasia with mental retardation syndrome (HPMRS) and to distinguish them from another subset of GPIBDs, termed multiple congenital anomalies hypotonia seizures syndrome (MCAHS). However, the increasing number of individuals with a GPIBD shows that hyperphosphatasia is a variable feature that is not ideal for a clinical classification.

**Methods:**

We studied the discriminatory power of multiple GPI-linked substrates that were assessed by flow cytometry in blood cells and fibroblasts of 39 and 14 individuals with a GPIBD, respectively. On the phenotypic level, we evaluated the frequency of occurrence of clinical symptoms and analyzed the performance of computer-assisted image analysis of the facial gestalt in 91 individuals.

**Results:**

We found that certain malformations such as Morbus Hirschsprung and diaphragmatic defects are more likely to be associated with particular gene defects (*PIGV*, *PGAP3*, *PIGN*). However, especially at the severe end of the clinical spectrum of HPMRS, there is a high phenotypic overlap with MCAHS. Elevation of AP has also been documented in some of the individuals with MCAHS, namely those with *PIGA* mutations. Although the impairment of GPI-linked substrates is supposed to play the key role in the pathophysiology of GPIBDs, we could not observe gene-specific profiles for flow cytometric markers or a correlation between their cell surface levels and the severity of the phenotype. In contrast, it was facial recognition software that achieved the highest accuracy in predicting the disease-causing gene in a GPIBD.

**Conclusions:**

Due to the overlapping clinical spectrum of both HPMRS and MCAHS in the majority of affected individuals, the elevation of AP and the reduced surface levels of GPI-linked markers in both groups, a common classification as GPIBDs is recommended. The effectiveness of computer-assisted gestalt analysis for the correct gene inference in a GPIBD and probably beyond is remarkable and illustrates how the information contained in human faces is pivotal in the delineation of genetic entities.

**Electronic supplementary material:**

The online version of this article (doi:10.1186/s13073-017-0510-5) contains supplementary material, which is available to authorized users.

## Background

Inherited deficiencies of glycosylphosphatidylinositol (GPI) biosynthesis are a heterogeneous group of recessive Mendelian disorders that all share a common feature: the function of GPI-linked proteins is compromised due to a defect in GPI anchor synthesis or modification. Most of the enzymes involved in this molecular pathway are known and the biochemical steps are well described [1]. With respect to the effect of genetic mutations on the anchor and the GPI-linked substrate, several subdivisions of the pathway have been in use: 1) early GPI anchor synthesis, 2) late GPI anchor synthesis, 3) GPI transamidase, and 4) remodeling of fatty acids of the GPI anchor after attachment to proteins (Additional file 1: Figure S1).

The last two groups are defined by their molecular actions and comprise the genes *GPAA1*, *PIGK*, *PIGU*, *PIGS*, and *PIGT* for the GPI-transamidase and *PGAP1*, *PGAP2*, *PGAP3*, *MPPE1*, and *TMEM8* for fatty acid remodeling. The differentiation between early and late GPI anchor synthesis considers the molecular consequence of the glycosylphosphatidylinositol biosynthesis defect (GPIBD), which was suggested after an important finding from Murakami et al. [2] regarding the release of alkaline phosphatase (AP), a GPI anchor marker: if anchor synthesis is stuck at an earlier step, the transamidase is not activated and the hydrophobic signal peptide of GPI anchor substrates is not cleaved. As soon as the first mannose residue on the GPI anchor has been added by PIGM, the transamidase tries to attach the substrate to the anchor. However, if subsequent steps are missing, the GPI anchored proteins (GPI-APs) might be less stable and hyperphosphatasia was hypothesized to be a consequence thereof.

The activity of the AP was regarded as such a discriminatory feature that it resulted in the phenotypic series of hyperphosphatasia with mental retardation syndrome (HPMRS) 1 to 6, currently comprising the six genes *PGAP2*, *PGAP3*, *PIGV*, *PIGO*, *PIGW*, and *PIGY* [3–9]. Whenever a pathogenic mutation was discovered in a new gene of the GPI pathway and the developmentally delayed individuals showed elevated AP in the serum, the gene was simply added to this phenotypic series. If hyperphosphatasia was missing, the gene was linked to another phenotypic series, multiple congenital anomalies hypotonia seizures (MCAHS), that currently consists of *PIGA*, *PIGN*, and *PIGT* [10–12]. However, the convention of dividing newly discovered GPIBDs over these two phenotypic subgroups is only reasonable if they really represent distinguishable entities. This practice is now challenged by a growing number of exceptions. The expressivity of most features is variable and even the AP seems to be a biomarker with some variability: some individuals with mutations in *PIGA* also show elevated AP levels [10, 13–15], and some individuals with mutations in *PIGO*, *PIGW*, *PGAP2*, and *PGAP3* show AP levels that are only borderline high [16–20].

Recently, deleterious mutations were identified in *PIGC*, *PIGP*, and *PIGG* in individuals with intellectual disability (ID), seizures, and muscular hypotonia, but other features considered to be a prerequisite for MCAHS or HPMRS were missing [21–23]. Despite the large phenotypic overlap with most GPIBDs, a flow cytometric analysis of granulocytes in individuals with *PIGG* mutations did not show reduced surface levels for GPI-APs [21–23]. However, Zhao et al. [24] showed that an impairment of PIGG in fibroblasts affects the marker expression, indicating that there might also be variability depending on the tissue. In concordance with these finding, a case report of an individual with ID and seizures that has mutations in *PIGQ* seems suggestive of a GPIBD in spite of negative FACS results [25].

The work of Makrythanasis et al. [23] can also be considered as a turning point in the naming convention of phenotypes that are caused by deficiencies of the molecular pathway as OMIM has now started referring to them as GPIBDs (see OMIM entry #610293 for a discussion). In this work we go one step further in this direction and ask whether the phenotypic series MCAHS and HPMRS should also be abandoned in favor of a more gene-centered description of the phenotype, which would also be in accordance with what Jaeken [26] already suggested for other congenital disorders of glycosylation. Referring to GPIBD phenotypes in a gene-specific manner makes particular sense if the gene can be predicted from the phenotypic level with some accuracy. For this purpose, we systematically analyzed the discriminatory power for GPIBDs for previously reported individuals as well as 23 novel cases that were identified through routine diagnostics. This also adds novel FACS results for 16 patients (blood or fibroblasts) as well as 19 novel mutations (Additional file 1: Figure S2).

Apart from founder effects that explain the reoccurrence of certain mutations at higher frequency, pathogenic mutations have now been reported in many exons (Additional file 1: Figure S2). However, not much is known about genotype–phenotype correlations in these genes, which makes bioinformatics interpretation of novel variants challenging. The phenotypic analysis, for which we received ethics approval from the Charité University and obtained informed consent from the responsible persons on behalf of all study participants, is based on three different data sources: 1) a comprehensive clinical description of the phenotypic features in human phenotype ontology terminology [27]; 2) flow cytometric profiles of multiple GPI-linked markers; and 3) computer-assisted pattern recognition on frontal photos of individuals with a molecularly confirmed diagnosis.

The rationale behind flow cytometry and image analyses is that GPIBDs might differ in their effect on GPI-APs and their trafficking pathways, resulting in distinguishable phenotypes. Interestingly, we found that the facial gestalt was well suited for delineating the molecular entity. The high information content of facies has become accessible recently through advanced phenotypic tools that might also be used for the analysis of other pathway disorders. Before we present the flow cytometry and automated image analysis results we review the most important phenotypic features of GPIBDs in the old schema of phenotypic series HPMRS and MCAHS.

## Methods

### Clinical overview of HPMRS

HPMRS, which is also sometimes referred to as Mabry syndrome (HPMRS1-6: MIM 239300, MIM 614749, MIM 614207, MIM 615716, MIM 616025, MIM 616809), can present as an apparently non-syndromic form of ID at one end of the clinical spectrum but also as a multiple congenital malformation syndrome at the other end (Table 1). The distinct pattern of facial anomalies of Mabry syndrome consist of wide set eyes, often with a large appearance and upslanting palpebral fissures, a short nose with a broad nasal bridge and tip, and a tented upper lip. The results of a computer-assisted comparison of the gene-specific facial gestalt is given in the “Comparison of the facial gestalt of GPIBDs” section.

**Table 1:** Summary of clinical findings in patients carrying *PIGV*, *PIGO*, *PGAP2*, *PGAP3*, *PIGW*, and *PIGY* mutations

	HPMRS1 *PIGV* (*n* = 26, excluding 2 fetus)	HPMRS2 *PIGO* (*n* = 16)	HPMRS3 *PGAP2* (*n* = 12)	HPMRS4 *PGAP3* (*n* = 26)	HPMRS5 *PIGW* (*n* = 3)	HPMRS6 *PIGY* (*n* = 4)
Hyperphosphatasia	26/26	14/14, ND in 2	6/6, ND in 6	25/26	1/3	4/4
Growth parameters						
OFC	Normal in 22/26 (microcephaly in 2/26, macrocephaly in 2/26)	Normal in 2/6 (microcephaly in 4, macrocephaly in 2, ND in 8)	Normal in 5/12 (microcephaly in 7)	Normal in 17/26 (microcephaly in 7, macrocephaly in 2)	Normal in 2 (ND in 1)	Normal in 2/4 (microcephaly in 2)
Height	Normal in 24/26	Normal in 3/5 (short stature in 2, ND in 11)	Normal in 2/2, ND in 10	Normal in 25/26 (short stature in 1/26)	Normal in 2 (ND in 1)	Normal in 2/4 (short stature in 2/4)
Weight	Normal in 24/26	Normal in 4/5 (dystrophy in 1, ND in 11)	Normal in 2/2, ND in 10	Normal in 21/26 (overweight in 2/26, dystrophy in 3/26)	Normal in 2 (ND in 1)	ND
Neurological phenotype						
Global developmental delay	26/26	16/16	2/2	26/26	3/3	4/4
Motor delay	26/26	16/16	12/12 (mild in 5)	26/26	3/3	4/4
Speech and language developmental delay	26/26 (no speech in 6/10)	16 (no speech in 5/16)	11/12	26/26 (no speech in 20/26)	3/3	4/4
Muscular hypotonia	18/24, ND in 2	11/11, ND in 5	5/6, ND in 6	23/26	2/2, ND in 1	ND
Seizures	20/26	11/12, ND in 4	8/12	17/26	Autistic traits 1/3	2/4
Behavioral abnormalities	ND	ND	ND	21/26	ND	2/4
Other neurological abnormalitites	Hearing loss	Hearing impairment (5/16), thin corpus callosum	Hearing impairment	Ataxia (10/26); no walking in 8/26	-	Regression of acquired skills (2/4)
	HPMRS1 (PIGV)	HPMRS2 (PIGO)	HPMRS3 (PGAP2)	HPMRS4 (PGAP3)	HPMRS5 (PIGW)	HPMRS6 (PIGY)
Malformations						
Cleft palate	8/26	4/16	1/12	15/26	-	0/4
Megacolon	8/26	5/16	1/12	0/26	-	0/4
Anorectal malformations	9/26	3/16	1/12	0/26	-	0/4
Vesicoureteral/renal malformations	6/10	2/16	ND	0/26	-	1/4
Heart defect	5/26	2/16	2/12	2/26	-	0/4
Facial gestalt						
Apparent hypertelorism	26/26	6/6, ND in 10	1/12	12/13, ND in 13	ND	1/4
Up-slanting palpebral fissure	26/26	10/11, ND in 5	ND in 12	2/26	ND	0/4
Broad nasal bridge	26/26	5/6, ND in 10	2/12	13/13, ND in 13	1/3 ND in 2	1/4
Broad nasal tip	26/26	5/6, ND in 10	1/12	4/14, ND in 12	ND	1/4
Short nose	26/26	5/6, ND in 10	1/12	14/24, ND in 2	ND	ND
Tented upper lip vermilion	26/26	7/8, ND in 8	2/12	17/24, ND in 21	3/3	ND
Large, fleshy ear lobes	-	1/16		18/24, ND in 21	ND	4/4
Brachytelephalangy	26/26	10/10, ND in 6	0/12 (broad nails in 1/12)	0/26 (broad nails in 6/26)	-	1/4
Further anomalies (rare)	Gastroesophageal reflux, optic atrophy bilateral, scoliosis, hip subluxation (right), thin corpus callosum, gingiva hyperplasia	Coronal synostosis, keratoderma, micrognathia, auricular malformations		Thin corpus callosum (9/26), ventriculomegaly (3/26), vermis hypoplasia (4/26)	Inguinal hernia (1/3)	Cataracts (2/4)
						Rhizomelic shortness of limbs (2/4)
						Contractures (2/4)
						Hip dysplasia (2/4)
Published cases	Rabe et al. 1991 [33]	Krawitz et al. 2012 [7]	Hansen et al. 2013 [4]	Howard et al. 2014 [5]	Chiyonobu et al. 2014 [3]	Ilkovski et al. 2015 [6]
	Marcelis et al. 2007 [34]	Kuki et al. 2013 [36]	Krawitz et al. 2013 [8]	Knaus et al. 2016 [19]	Hogrebe et al. 2016 [20]	
	Krawitz et al. 2010 [9] and Horn et al. 2010 [60]	Nakamura et al. 2014 [16]	Jezela-Stanek et al. 2016 [18]	Pagnamenta et al. 2017 [40]		
	Horn et al. 2011 [28]	Xue et al. 2016 [31]	Naseer et al. 2016 [38]	Nampoothiri et al. 2017 [41]		
	Thompson et al. 2012 [29]	Morren et al. 2017 [35]		Abdel-Hamid et al. 2017 [39]		
	Horn et al. 2014 [30]	Zehavi et al. 2017 [17]		2 unpublished cases		
	Xue et al. 2016 [31]	Tanigawa 2017 [37]				
	Reynolds et al. 2017 [32]					
	6 unpublished cases					

*ND* not documented; *OFC* occipitofrontal head circumference

Psychomotor delay, ID, and variable AP elevation are the only consistent features of all individuals with pathogenic mutations in *PIGV* [9, 28–34], *PIGO* [7, 16, 17, 31, 35–37], *PGAP2* [4, 8, 18, 38], *PGAP3* [5, 19, 39–41], and *PIGY* [6]. Speech development, especially expressive language, is more severely affected than motor skills in the majority of the affected individuals (Table 1). Absent speech development was observed in more than half of the affected individuals. Speech difficulties may be complicated by hearing loss, which is present in a minority of affected individuals. In the different genetic groups, seizures of various types and onset are present in about 65% of affected individuals. Most affected individuals show a good response to anticonvulsive drugs, but a few affected individuals are classified as drug resistant and represent the clinically severe cases (individual 14-0585). Muscular hypotonia is common in all types of HPMRS (about 85%). Behavioral problems, in particular sleep disturbances and autistic features, tend to be frequent (81%) in affected individuals with *PGAP3* mutations and are described in a few affected individuals with *PIGY* mutations but are not documented in affected individuals with mutations in the other four genes. Furthermore, ataxia and unsteady gait have been documented in almost half of the affected individuals carrying *PGAP3* mutations and about a third of this group did not achieve free walking at all.

Elevated values of AP were the key finding in affected individuals. However, a few cases are documented with only minimal elevation of this parameter. The degree of persistent hyperphosphatasia in the reported affected individuals varies over a wide range between about 1.1 and 17 times the age-adjusted upper limit of the normal range. The mean elevation of AP is about five to six times the upper limit. Measurements at different ages of one individual show marked variability of this value, for example, from two to seven times the upper limit. There is no association between the AP activity and the degree of neurological involvement. Furthermore, there is no correlation between the mutation class and genes with the level of elevation of AP.

Growth parameters at birth are usually within the normal range. Most affected individuals remain in the normal range, although there is evidence of a skewed distribution towards the upper centiles and a few affected individuals become overweight. In contrast, about 8% of the affected individuals develop postnatal short stature and fail to thrive. About 28% of affected individuals develop microcephaly, whereas less than 10% become macrocephalic. Abnormalities of growth and head size do not correlate with a specific mutation or gene within this group of genes.

Involvement of other organ systems varies among the genetically different groups. *PIGV*, *PIGO*, and *PGAP2* affected individuals frequently suffer from a variety of different malformations. Anorectal malformations, such as anal atresia or anal stenosis, are the most frequent anomalies with almost 30% penetrance in the group of affected individuals. The second most frequent anomaly is Hirschsprung disease with a frequency of about 26% in the same group of affected individuals. Vesicoureteral or renal malformations occur with a similar frequency; among these are congenital hydronephrosis, megaureter, and vesicoureteral reflux. Our data revealed a frequency of heart defects of about 20% in the group of affected individuals with *PIGV*, *PIGO*, and *PGAP2* mutations; however, the type of cardiac abnormality is variable. Only 2 of 26 affected individuals carrying *PGAP3* mutations have variable congenital heart defects. Cleft palate is the malformation with the highest frequency in the group of affected individuals with *PGAP3* mutations with a prevalence of almost 60%, whereas other malformations are rarely observed. Exceptional is a group of ten Egyptian individuals with the same founder mutation and a high incidence of structural brain anomalies (thin corpus callosum (8/10), vermis hypoplasia (4/10), ventriculomegaly (3/10), and Dandy-Walker malformation (1/10)) [39]. To date these are the few individuals with a presumably complete loss of function for this gene (NM_033419.3:c.402dupC, p.Met135Hisfs*28; c.817_820 delGACT, p.Asp273Serfs*37).

Malformations had not been observed in the single reported affected individual with *PIGW* mutations [3]. Apart from dilation of renal collecting systems, affected individuals with *PIGY* mutations presented with a new spectrum of organ involvement such as cataracts, rhizomelic shortness of limbs, contractures and hip dysplasia [6].

All affected individuals with *PIGV* and *PIGO* mutations had a variable degree of distal hand anomalies, namely brachytelephalangy. They showed hypoplastic finger nails as well as hypoplastic distal phalanges in hand X-rays. Often, they displayed broad and short distal phalanges of the thumbs and halluces, including short and broad corresponding nails of the affected digits. Brachytelephalangy is not present in any of the affected individuals with *PGAP3*, *PGAP2*, and *PIGW* mutations, respectively, although one-third showed broad nails without radiological abnormalities in the available X-rays. One of four affected individuals with *PIGY* mutations showed brachytelephalangy.

A multidisciplinary approach is required to manage the GPIBDs described in this section, as the clinical variability is broad. It is recommended that all affected individuals have at least one baseline renal ultrasound investigation as well as echocardiography to rule out any obvious malformations. In case of chronic obstipation, Hirschsprung disease, as well as anal anomalies, should be excluded. Hearing evaluation is recommended in all affected individuals. Individuals with behavioral problems may benefit from a review by a clinical psychologist. Regular developmental assessments and EEG investigations are required to ensure that affected individuals get optimal support. The tendency towards epilepsies has been reported to decrease in some affected individuals with age and if the affected individual and physician agree to a trial discontinuation of therapy, medications could be tapered.

### Clinical overview of MCAHS

MCAHS comprises a group of genetically different disorders characterized by early onset forms of different types of epilepsies with poor prognosis, missing or minimal psychomotor development, and often, early death (Table 2). The phenotypic series include individuals with *PIGA* (MIM 300868) [10, 13–15, 42–46], *PIGN* (MIM 614080) [12, 18, 47–53], and *PIGT* (MIM 615398) [11, 40, 54–57] mutations.

**Table 2:** Comparison of phenotypic data and biomarkers in different types of MCAHS

GPIBDs: affected gene (individuals)	MCAHS2 PIGA (*n* = 26)	MCAHS1 PIGN (*n* = 20, including three fetuses)	MCAHS3 PIGT (*n* = 14)
Hyperphosphatasia	+/−	+/−	+
Seizures with early onset	+	+	+
Early death	+/−	+/−	−
Profound ID	+	+	+
Neonatal muscular hypotonia	+/−	+/−	+
Macrocephaly or macrosomia	+/−	+/−	+/−
Variable brain anomalies	+/−	+	+
Hyperreflexia/contractures	+/−	+/−	ND
Variable facial anomalies	+/−	+/−	+/−
Renal/vesicoureteral anomalies	+/−	+/−	+/−
Gastrointestinal anomalies	+/−	+/−	ND
Cardiovascular abnormalities	ND	+/−	ND
Cleft palate	+	+/−	−
Diaphragmatic defect	−	+/−	−
Short distal phalanges	−	+/−	−
Elevated alkaline phosphatase (AP)	+/− (5/23 elevated AP)	−	Decreased AP
Abnormal flow cytometry results	+/ND	+/ND	+/ND
Published cases	Johnston et al. 2012 [10]	Maydan et al. 2011 [12]	Kvarnung 2013 [11]
	van der Crabben et al. 2014 [15]	Brady et al. 2014 [48]	Nakashima 2014 [54]
	Swoboda et al. 2014 [43]	Ohba et al. 2014 [47]	Lam 2015 [55]
	Kato et al. 2014 [14]	Couser et al. 2015 [49]	Skauli 2015 [56]
	Belet et al. 2014 [42]	Fleming et al. 2015 [52]	Kohashi 2017 [57]
	Tarailo-Graovac et al. 2015 [44]	Khayat et al. 2015 [53]	Pagnamenta 2017 [40]
	Joshi et al. 2016 [46]	Nakagawa et al. 2016 [50]	3 unpublished cases
	Fauth et al. 2016 [13]	Jezela-Stanek et al. 2016 [18]	
	Kim et al. 2016 [45]	McInerney-Leo et al. 2016 [51]	
	9 unpublished cases		

*ND* not documented

Neonatal muscular hypotonia is often present. The variable congenital anomalies affect the renal/vesicoureteral, cardiac, and gastrointestinal systems. Brain imaging showed variable abnormalities, for example, thin corpus callosum, cerebellar atrophy/hypoplasia, cerebral atrophy, and delayed myelination, but also normal findings in other affected individuals. There is overlap with the spectrum of malformations seen in HPMRS. Exceptions are megacolon, which is only reported in individuals with *PIGV*, *PIGO*, and *PGAP2* mutations, and diaphragmatic defects, which are only documented in three fetuses with *PIGN* mutations [51]. In addition, joint contractures and hyperreflexia are documented in some individuals with *PIGA* and *PIGN* mutations [10, 13–15, 42–46]. Macrocephaly or macrosomia occur in some of these individuals, whereas microcephaly occurs in others. No distinct facial phenotype is recognizable in comparison within and between the genetically different groups of MCAHS.

Interestingly, 5 out of 23 individuals with *PIGA* mutations had elevated AP measurements, whereas only one individual with *PIGN* mutations was reported with borderline high AP activity [52]. In contrast, some of the individuals with *PIGT* mutations showed decreased AP [11, 54].

HPMRS and MCAHS display an overlapping clinical spectrum but MCAHS has a considerably worse prognosis due to early onset and often intractable seizures as well as early death in the majority of affected individuals. However, facial dysmorphisms do not appear to be characteristic in the different types of MCAHS in contrast to HPMRS. Importantly, elevation of AP and reduced surface levels of GPI-linked substrates are not restricted to HPMRS.

### Flow cytometry

#### Flow cytometry analysis of blood

Flow cytometry was performed on granulocytes extracted from peripheral blood draws that were sampled in BCT CytoChex tubes (Streck, NE, USA), shipped, and analyzed in less than 72 h. Whole blood (50 μl) was mixed with 20 μl of an antibody panel:

1. 4 μl CD55-PE (BD #555694), 4 μl CD59-FITC (BD #555763), 2 μl CD45-PacBlue (Beckman Coulter, clone J.33), and 10 μl FACS buffer.
2. 2 μl CD16-PE (Beckman Coulter, clone 3G8), 4 μl FLAER-AF488 (FL2S-C; Burlington, Canada,), 2 μl CD45-PacBlue (Beckman Coulter, clone J.33), and 12 μl FACS buffer.
3. 2 μl CD24-APC (MiltenyiBiotec Clone REA832), 2 μl CD45-PacBlue (Beckman Coulter, clone J.33), and 16 μl FACS buffer.

The staining was incubated for 30 min at room temperature followed by an incubation with 500 μl red blood cell lysis buffer for 10 min. Debris was removed by discarding the supernatant after centrifugation and the cell pellet was washed twice with 200 μl and resuspended in 100 μl FACS buffer for flow cytometry analysis on a MACSQuant VYB (MiltenyiBiotec, Bergisch Gladbach, Germany).

Gating for living cells was based on forward and side scatter (FSC-A vs. SSC-A). Single cells were gated on a diagonal (FSC-A vs. FSC-H). Granulocytes were identified as granular (SSC-A high) and CD45-positive cells.

The reduction of GPI-AP expression was assessed by the ratio of the median fluorescence intensity (MFI) of the patient to the MFI of a shipped healthy control. Heterozygous carriers of pathogenic mutations (parents) were used as controls when unrelated healthy controls were not available. It is noteworthy that differences in GPI-AP expression were subtle in healthy parents compared to unrelated controls. To compare marker reduction of published and unpublished cases only FLAER and CD16 were used.

#### Flow cytometric analysis of fibroblast cells

Fibroblasts derived from skin biopsies of patients, parents, and healthy control individuals were cultured in DMEM supplemented with 10% FCS, 1% ultraglutamine, 1% penicillin/streptomycin. For flow cytometry analysis confluently grown cells were washed twice with PBS (-Ca^2+^, -Mg^2+^); the cells were gently detached from the coulter dish with Trypsin-EDTA (0.01%). The single cell suspension was washed with FACS buffer, counted, diluted (100.000 cells/stain), and centrifuged, after which the supernatant was discarded and the cell pellet was resuspended in the following antibody mix.

1. 4 μl CD55-PE (BD #555694), 4 μl CD59-FITC (BD #555763), and 12 μl FACS buffer.
2. 4 μl CD73-PE (BD#550257), 4 μl FLAER-AF488 (Cedarlane, FL2S-C), and 12 μl FACS buffer.

The staining was incubated for 30 min at room temperature followed by two washing steps with 200 μl FACS buffer. For flow cytometry analysis on a MACSQuant VYB the cells were resuspended in 100 μl FACS buffer.

Reduction of GPI-AP expression was calculated as a ratio between the median fluorescence intensity (MFI) of the patient against the mean of MFIs from healthy parents and a healthy unrelated control. It is noteworthy that heterozygous carriers of pathogenic mutations (parents) and unrelated healthy controls had only subtle differences in GPI-AP expression.

### Computer-assisted phenotype comparison

Facial images of all individuals with a molecularly confirmed GPIBD were assessed with the Face2Gene Research Application (FDNA Inc., Boston MA, USA). This software tool set allows the phenotypic comparison of user-defined cohorts with ten or more individuals. The classification model of Face2Gene Research uses a neural network architecture that consists of ten convolutional layers, each but the last followed by batch normalization. The original collections are split into training and test sets for cross-validation and mean accuracies for the classification process are computed. The result of a single experiment is a confusion matrix that describes the performance of the classification process. As cohort size is a known confounder, we randomly sampled all cohorts down to the same size (*n* = 10) and computed the mean true positive and error rates as well as the standard deviation from ten iterations [58]. The scripts for the simulations are available on request and can be used to reproduce the results.

## Results

### Flow cytometric assessment of GPIBDs

**Fig. 1:**
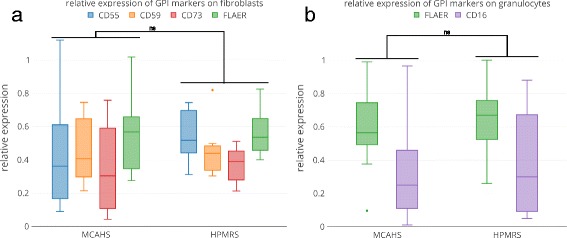
Flow cytometric profiling for GPIBDs. Cell surface levels of FLAER and tissue-specific GPI-anchored proteins were assessed on fibroblasts (**a**) as well as on granulocytes (**b**) of individuals affected by GPIBDs. The relative expression was grouped for GPIBDs of the same phenotypic series, MCAHS (*PIGA*, *PIGN*, *PIGT*) and HPMRS (*PGAP3*, *PIGV*, *PIGO*, *PIGW*), but showed no significant differences (significance was tested with Wilcoxon-Mann-Whitney test; the *p* value was corrected for sample size (Bonferoni))

We acquired fibroblast cultures of affected individuals to perform measurements under the same experimental conditions repeatedly. The marker FLAER, which binds to the GPI anchor directly, as well as the GPI-APs CD55, CD59, and CD73, which show high expression levels on fibroblasts, were assessed (Fig. 1). We hypothesized that measuring cell surface levels of GPI-linked substrates directly by flow cytometry might be more suitable to quantify the severity of a GPIBD or to predict the affected gene. No significant differences between patients with MCAHS were observed compared to patients with HPMRS (Fig. 1a). Furthermore, the cell surface levels of CD55 and CD59 were on average lower in cells that were derived from individuals with mutations in *PGAP3* compared to individuals with mutations in *PIGV* (Additional file 1: Table S1), although this did not correspond to a higher prevalence of seizures or a more severe developmental delay. CD55 and CD59 are of particular interest as they protect cells from an attack on the activated complement system and the membrane attack complex that has also been shown to be involved in the pathogenesis of seizures [59].

The samples with pathogenic mutations in *PIGV* are noteworthy as they are derived from individuals that differ considerably in the severity of their phenotype: 14-0585 was born with multiple malformations and his seizures are resistant to treatment, whereas the other three individuals, A2, A3, and P1, are considered to be moderately affected. The flow cytometric profiles, however, do not show marked differences. Furthermore, the cell surface levels of CD55 and CD59 were on average lower in cells that were derived from individuals with mutations in *PGAP3*.

While the reproducibility of the flow cytometric data on fibroblasts is attractive, the small size of the sample set is clearly a disadvantage in the assessment of potential differences between the phenotypic subgroups of GPIBDs. Most flow cytometric analyses have been performed on granulocytes of affected individuals with the markers CD16 and FLAER and we added a comparison of the relative median fluorescent intensities (rMFI) for a total of 39 individuals of the phenotypic series MCAHS and HPMRS (Additional file 1: Table S2). Although individuals of the MCAHS spectrum are usually more severely affected than individuals of the HPMRS spectrum, we did not observe any significant differences for the tested markers (Fig. 1b). Thus, no significant correlation between FACS profiles of the two phenotypic series was found.

### Comparison of the facial gestalt of GPIBDs

The craniofacial characteristics of many Mendelian disorders are highly informative for clinical geneticists and have also been used to delineate gene-specific phenotypes of several GPIBDs [3–5, 10, 12, 19, 28–30, 32, 39, 40, 43, 44, 60, 61]. However, our medical terminology is often not capable of describing subtle differences in the facial gestalt. Therefore, computer-assisted analysis of the gestalt has recently received much attention in syndromology and several groups have shown that the clinical face phenotype space (CFPS) can also be exploited by machine learning approaches [62]. If a recognizable gestalt exists, a classifier for facial patterns can be trained to infer likely differential diagnoses. Conversely, if photos of affected individuals with disease-causing mutations in different genes of a pathway form separate clusters, it indicates that the gestalt is distinguishable to a certain extent. FDNA’s recently launched RESEARCH application is a deep learning tool for exactly this purpose (https://app.face2gene.com/research): a classification model is generated on two or more collections of frontal images and the performance is reported as a confusion matrix. If true positive rates for the single gene–phenotypes are achieved that are significantly better than for a random assignment of photos to cohorts, there is some phenotypic substructure and the null hypothesis of perfect heterogeneity may be rejected.

**Fig. 2:**
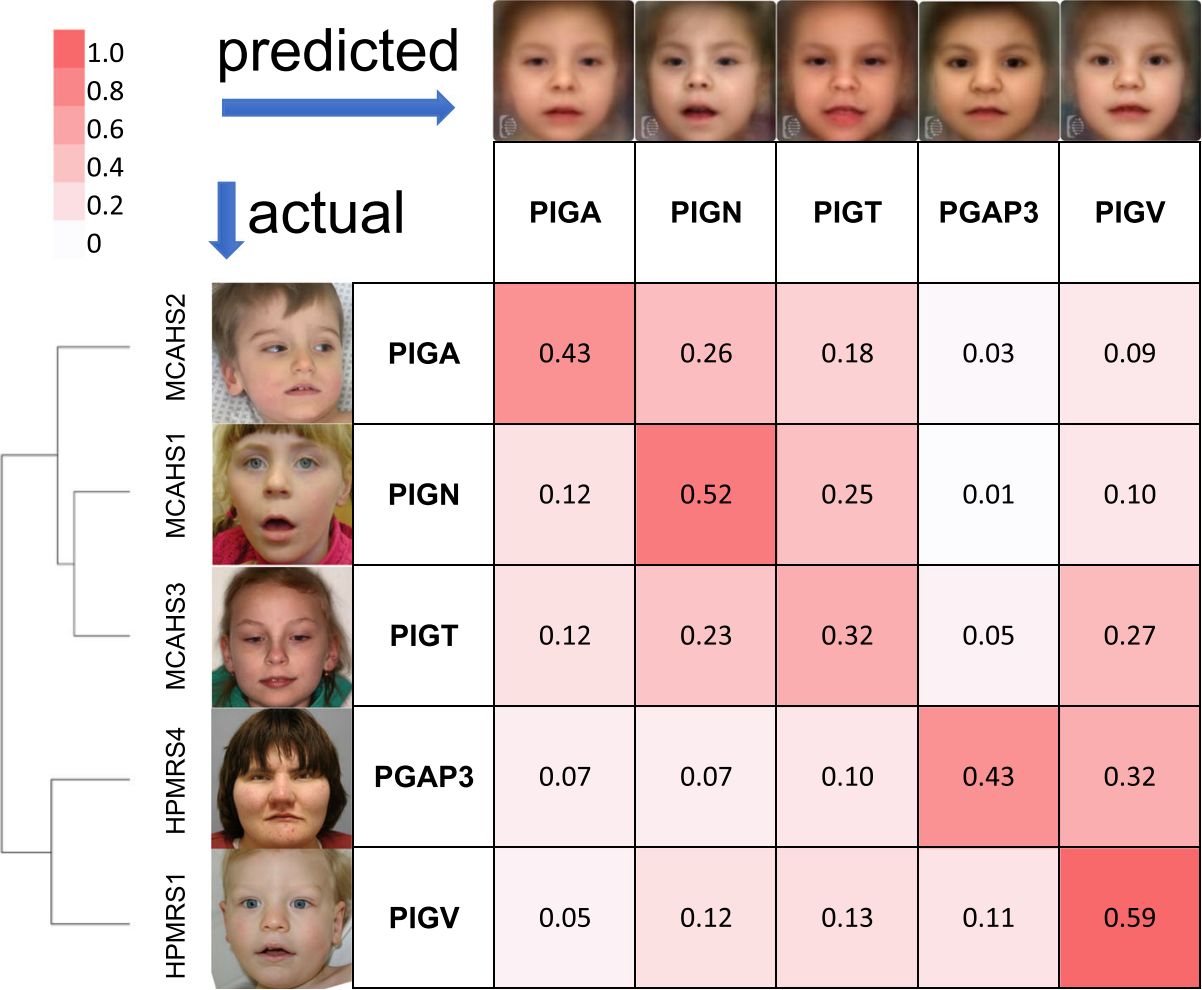
Automated image analysis for five of the most prevalent GPIBDs. A model for the classification of the gene–phenotypes was repeatedly trained and cross-validated on patient subsets that were randomly down-sampled to the same cohort size of n = 10. A mean accuracy of 0.44 was achieved, which is significantly better than random (0.20). For explanatory purposes, the rows of the confusion matrix start with instances of previously published or newly identified individuals with GPIBDs. If the predicted gene matches the molecularly confirmed diagnosis, such a test case would contribute to the true positive rate, shown on the diagonal. Actual affected individual photographs were used to generate an averaged and de-identified composite photo and are shown at the top of the columns. The performance of computer-assisted image classification is significantly better than expected under the null model of perfect heterogeneity and indicates a gene-specific phenotypic substructure for the molecular pathway disease. Higher false positive error rates occur between genes of the same phenotypic series, HPMRS and MCAHS, as indicated by the dendrogram

We used the RESEARCH app of the Face2Gene suite to evaluate a classifier for the five most prevalent GPIBDs, *PIGA* (*n* = 20), *PIGN* (*n* = 11), *PIGT* (*n* = 12), *PIGV* (*n* = 25), and *PGAP3* (*n* = 23). Our original sample set thus consists of frontal facial photos of 91 individuals with a molecularly confirmed diagnosis of HPMRS or MCAHS, including cases that have been previously published [5, 9–11, 13–15, 19, 28–30, 32–34, 39, 43, 47, 49, 50, 52–56, 60, 63]. The mean accuracy that was achieved on this original sample set was 52.2%, which is significantly better than random. In order to compare the performance for the single gene classes we had to exclude confounding effects from unbalanced cohort sizes and sampled the cohorts down to the same size of *n* = 10. Although this decreases the overall performance, the mean accuracy of 45.8% is still significantly better than the 20% that would be achieved by chance in a five-class problem for evenly sized cohorts (Fig. 2). Furthermore, for every single gene–phenotype, the true positive rate (TPR) was better than randomly expected, with *PIGV* achieving the highest value (59%).

Interestingly, we observed the highest false negative rate in the confusion matrix for *PGAP3* (HPMRS4): on average 32% of these cases are erroneously classified as *PIGV* (HPMRS1) cases. This finding is in good agreement with the phenotypic delineation from syndromologists that grouped these to genes in the same subclass. A cluster analysis of the confusion matrix actually reproduces the two phenotypic series as shown by the dendrogram in Fig. 2.

While the confusion matrix on the entire sample set can be used to decide whether there are gene-specific substructures in the GPI pathway, pairwise comparisons are better suited to derive phenotypic differences between genes even inside a phenotypic series. We therefore evaluated the area under the receiver operating characteristics curve (AUC) and found the correct gene prediction more often than randomly expected, including *PIGV* versus *PGAP3* (Additional file 1: Figure S3). The differences in pair-wise comparison between *PIGV* and *PGAP3* could be confounded by the large number of Egyptian cases in the *PGAP3* cohort [39], the effect of which we could not further analyze due to the limited set of patient photos.

## Discussion

The identification of multiple affected individuals with GPIBDs has enabled the analysis of genotype–phenotype relationships for the molecular pathway of GPI anchor synthesis. Besides a developmental delay and seizures, which are common findings in most affected individuals with a GPIBD, the clinical variability and the variation in expressivity is wide. So far, recognizable gene-specific phenotypes seem to be accepted for *PIGL* and are discussed for *PIGM* [64, 65]. For other GPIBDs the phenotypic series HPMRS and MCAHS have been used to subgroup the pathway and the activity of AP in serum was the main classification criterion. However, these disease entities are increasingly cumbersome as some cases are now known to not follow this oversimplified rule.

We therefore compared GPIBDs based on deep phenotyping data and flow cytometric profiles of GPI-APs. Among the 16 genes of the GPI pathway with reports of affected individuals, mutations in *PIGA*, *PIGN*, *PIGT*, *PIGV*, and *PGAP3* were most numerous and these GPIBDs were also suitable for automated image analysis.

A systematic evaluation of the phenotypic features showed that certain malformations occur with a higher frequency in specific GPIBDs. To date, megacolon has only been found to be associated with *PIGV*, *PIGO*, and *PGAP2* mutations. Diaphragmatic defects have only been documented in affected individuals with *PIGN* mutations. Only in individuals with *PGAP3* mutations are behavioral problems, especially sleep disturbances and autistic features, present, in about 90%. In addition, ataxia and unsteady gait are also frequently documented in this group but not in the others. An accurate classification that is merely based on clinical symptoms is, however, not possible due to their high variability. Also, flow cytometric analysis of GPI marker expression was not indicative for the gene defect and did not correlate with the severity of the phenotype. Of note, however, an assessment of the GPI-AP expression levels seems more sensitive in fibroblasts than in blood cells [24]. This might also be related to the trafficking pathways of GPI-APs through endoplasmic reticulum and Golgi that differ for cell types and substrates [66, 67].

The overlapping clinical spectrum of both HPMRS and MCAHS, the findings of elevated AP, and the reduced surface levels of GPI-linked proteins in some of the MCAHS cases favor a common classification as GPIBDs.

In light of the high variability and expressivity of the clinical findings and the weak genotype–phenotype correlation, the most surprising finding of our study was the high discriminatory power that facial recognition technology achieved. In spite of the similarity of the pathophysiology, differences in the gestalt are still perceptible. This illustrates the remarkable information content of human faces and advocates for the power of computer-assisted syndromology in the definition of disease entities.

Automated image analysis of syndromic disorders is a comparably new field of research and the approach that we used requires photos of at least ten individuals per cohort. However, it is currently not known if there is a minimum number of cases that is required to assess whether a gene–phenotype is recognizable. Furthermore, for every rare disorder with a characteristic gestalt there is possibly a maximum value for the recognizability. So far, the approximation of this upper limit has not systematically been studied depending on the number of individuals that were used in the training process and should definitely be a focus for future research.

## Conclusions

A gene-centered classification of GPIBDs is recommended due to the overlapping clinical spectrum of both HPMRS and MCAHS in the majority of affected individuals. Measuring AP serum activity and cell surface levels of GPI-linked markers is still regarded as an imperative in the diagnostic work-up of GPIBDs, especially when dealing with mutations that have not been reported previously. In addition, next-generation phenotyping tools can add another layer of information in the interpretation of novel mutations in the GPI anchor pathway, particularly if the flow cytometric data are inconclusive.

## Additional file

Additional file 1: Supplemental tables and figures. (DOCX 1270 kb)

## Abbreviations

AP: Alkaline phophatase
APC: Allophycocyanin
AUC: Area under the curve
DMEM: Dulbecco's modified Eagle medium
DPDL: Deep Phenotyping for Deep Learning
EEG: Electroencephalography
FACS: Fluorescence-activated cell sorting
FCS: Fetal calf serum
FITC: Fluorescein isothiocyanate
FLAER: Fluorescein-labeled proaerolysin
FSC-A: Forward scatter area
FSC-H: Forward scatter area hight
GPI: Glycosylphosphatidylinositol
GPI-AP: GPI-anchored protein
GPIBD: GPI biosynthesis defect
HPMRS: Hyperphosphatasia with mental retardation syndrome
ID: Intellectual disability
MCAHS: Multiple congenital anomalies hypotonia seizures
MFI: Media fluorescent intensity
OFC: Occipitofrontal head circumference
PBS: Phosphate buffered saline
PE: Phycoerythrin
SSC-A: Side scatter area
TPR: true positive rate
