# Supplementary material for: Characterization of glycosylphosphatidylinositol biosynthesis defects by clinical features, flow cytometry, and automated image analysis
Source: Genome Med. 2018 Jan 9;10:3. doi: 10.1186/s13073-017-0510-5 (PMC5759841; doi:10.1186/s13073-017-0510-5)
Supplement: Additional file 1: — Supplemental tables and figures. (DOCX 1270 kb) [file 13073_2017_510_MOESM1_ESM.docx]

Additional file 1

Table S1: Mean relative reduction of GPI marker (FLAER, CD55, CD59 and CD73) expression on fibroblasts. Mutations are with respect to the following transcripts: *PIGN*: NM_176787.4, *PIGT*: NM_015937.5, *PIGV*: NM_017837.3, *PGAP3*: NM_033419.3

| **Reference**  MCHAS1 | **Patient ID** | **Gene** | **CD55** | **CD59** | **CD73** | **FLAER** | **Mutation** |
| --- | --- | --- | --- | --- | --- | --- | --- |
| Maydan2011 | V9 | PIGN | 0,09 | 0,70 | 0,18 | 0,45 | c.2126G>A |
| Maydan2011 | V10 | PIGN | 0,33 | 0,75 | 0,61 | 0,67 | c.2126G>A |
|  |  |  |  |  |  |  |  |
| MCHAS2 |  |  |  |  |  |  |  |
| Knaus2017 | 17-1611 | PIGA | 0,36 | 0,29 | 0,08 | 0,31 | c.13+1G>C |
| Knaus2017 | 17-1612 | PIGA | 0,11 | 0,21 | 0,04 | 0,28 | c.13+1G>C |
|  |  |  |  |  |  |  |  |
| MCHAS3 |  |  |  |  |  |  |  |
| Knaus2017 | 15-2114 | PIGT | 0,38 | 0,31 | 0,30 | 0,57 | c.1472T>A, c.1484+2T>A |
| Knaus2017 | 17-0871 | PIGT | 0,69 | 0,41 | 0,52 | 0,63 | c.1582G>A |
| Knaus2017 | 17-0872 | PIGT | 1,12 | 0,50 | 0,76 | 1,02 | c.1582G>A |
|  |  |  |  |  |  |  |  |
|  |  |  |  |  |  |  |  |
| Krawitz2010  HPMRS1 | A2 | PIGV | 0,52 | 0,44 | 0,27 | 0,46 | c.1022C>A |
| Krawitz2010 | A3 | PIGV | 0,74 | 0,82 | 0,21 | 0,54 | c.1022C>A |
| Horn2011 | P1 | PIGV | 0,70 | 0,31 | 0,39 | 0,40 | c.467G>A, c.1022C>A |
| Knaus2017 | 14-0585 | PIGV | 0,70 | 0,50 | 0,51 | 0,46 | c.494C>A, c.1405C>T |
|  |  |  |  |  |  |  |  |
| HPMRS4 |  |  |  |  |  |  |  |
| Knaus2016 | A-II-1 | PGAP3 | 0,46 | 0,30 | 0,46 | 0,59 | c.320C>T, c.558-10G>A |
| Knaus2016 | B-II-1 | PGAP3 | 0,31 | 0,41 | 0,31 | 0,67 | c.402dupC, c.558-10G>A |
| Knaus2016 | C-II-2 | PGAP3 | 0,44 | 0,44 | 0,44 | 0,83 | c.861G>T, c.∗559C>T |

Table S2: Relative reduction of GPI marker (FLAER and CD16) expression on granulocytes from all published GPIBD cases. *PIGA*: NM_002641.3, *PIGN*: NM_176787.4, *PIGT*: NM_015937.5, *PIGV*: NM_017837.3, *PGAP3*: NM_033419.3, *PIGW*: NM_178517.4, *PIGO*: NM_032634.3.

| **Reference**  MCHAS1 | **Patient ID** | **Gene** | **FLAER** | **CD16** | **Mutation** |
| --- | --- | --- | --- | --- | --- |
| Ohba2014 | II_2 | PIGN | 0,79 | 0,26 | c.808T>C, c.963G>A |
| Ohba2014 | II_5 | PIGN | 0,65 | 0,33 | c.808T>C, c.963G>A |
| Khayat2016 | IV_E | PIGN | 0,53 | 0,96 | c.755A>T |
| Nakagawa2016 | P1 | PIGN | 0,70 | 0,96 | c.808T>C, del exon 2-14 |
|  |  |  |  |  |  |
| Kato2014  MCHAS2 | IV-2 | PIGA | 0,40 | 0,15 | c.1234C>T |
| Kato2014 | IV-2 | PIGA | 0,40 | 0,15 | c.1234C>T |
| Kato2014 | P3 | PIGA | 0,60 | 0,15 | c.230G>T |
| Kato2014 | P4 | PIGA | 0,60 | 0,09 | c.230G>T |
| Kato2014 | P2 | PIGA | 0,80 | 0,05 | c.616A>T |
| Kato2014 | P5 | PIGA | 0,80 | 0,12 | c.355C>T |
| Swoboda2013 | IV-3 | PIGA | 0,50 | 0,50 | c.1030_1032delCCT |
| Knaus2017 | 17-0311 | PIGA | 0,79 | 0,76 | c.229C>G |
| Knaus2017 | 17-0551 | PIGA | 0,38 | 0,42 | c.356G>A |
| Knaus2017 | 17-0661 | PIGA | 0,10 | 0,03022339 | c.565A>G |
| Knaus2017 | 17-0816 | PIGA | 0,53 | 0,32 | c.1354G>T |
| Knaus2017 | 17-1067 | PIGA | 0,49 | 0,01 | c.145G>A |
| Knaus2017 | 17-1592 | PIGA | 0,62 | 0,09 | c.242G>A |
| MCHAS3 |  |  |  |  |  |
| Kvarnung2013 | V1 | PIGT | 0,50 | 0,25 | c.547A>C |
| Kvarnung2013 | V2 | PIGT | 0,50 | 0,25 | c.547A>C |
| Kvarnung2013 | V5 | PIGT | 0,99 | 0,25 | c.547A>C |
| Nakashima2014 | P1 | PIGT | 0,48 | 0,54 | c.250G>T, c.1342C>T |
| Lam2015 | P1 | PIGT | 0,60 | 0,10 | c.918dupC, c.1342C>T |
| Lam2015 | P2 | PIGT | 0,82 | 0,27 | c.918dupC, c.1342C>T |
| Skauli2016 | P1 | PIGT | 0,48 | 0,19 | c.1079G>T |
| Knaus2017 | 16-2261 | PIGT | 0,49 | 0,59 | c.494-2A, c.1582G>A |
| HPMRS1 |  |  |  |  |  |
| Krawitz2010 | A2 | PIGV | 0,70 | 0,65 | c.1022C>A |
| Krawitz2010 | A3 | PIGV | 0,75 | 0,68 | c.1022C>A |
| Krawitz2010 | D | PIGV | 0,75 | 0,75 | c.1022C>A |
| Knaus2017 | 16-1253 | PIGV | 0,57 | 0,58 | c.265C>T, c.1022C>A |
| HPMRS2 |  |  |  |  |  |
| Knaus2017 | 15-1328 | PIGO | 0,59 | 0,42 | c.2191del, c.458T>C |
| Knaus2017 | 15-1329 | PIGO | 0,36 | 0,30 | c.2191del, c.458T>C |
| Kuki2013 | P1 | PIGO | 0,31 | 0,06 | c.355C>T, c.2497_2498del |
| Nakamura2014 | II_1 | PIGO | 1,00 | 0,71 | c.389C>A, c.1288C>T |
| Tanigawa2017 | BII-2 | PIGO | 0,26 | 0,05 | c.1109A>G, c.2496Tdel |
| Tanigawa2017 | CII-2 | PIGO | 1,00 | 0,17 | c.1031T>A, c.1228C>T |
| Tanigawa2017 | CII-3 | PIGO | 0,51 | 0,13 | c.1031T>A, c.1228C>T |
| Tanigawa2017  HPMRS4 | DII-1 | PIGO | 0,60 | 0,06 | c.1288C>T, c.3139A>G |
|  |  |  |  |  |  |
| Howard2014 | B-II-1 | PGAP3 | 0,67 | 0,30 | c.439dupC, c.914A>G |
| Knaus2016 | F-II-3 | PGAP3 | 0,89 | 0,88 | c.511T>C, c.842T>C |
| HPMRS5 |  |  |  |  |  |
| Chiyonobu2014 | P1 | PIGW | 0,76 | 0,08 | c.211A>C , c.499A>G, |


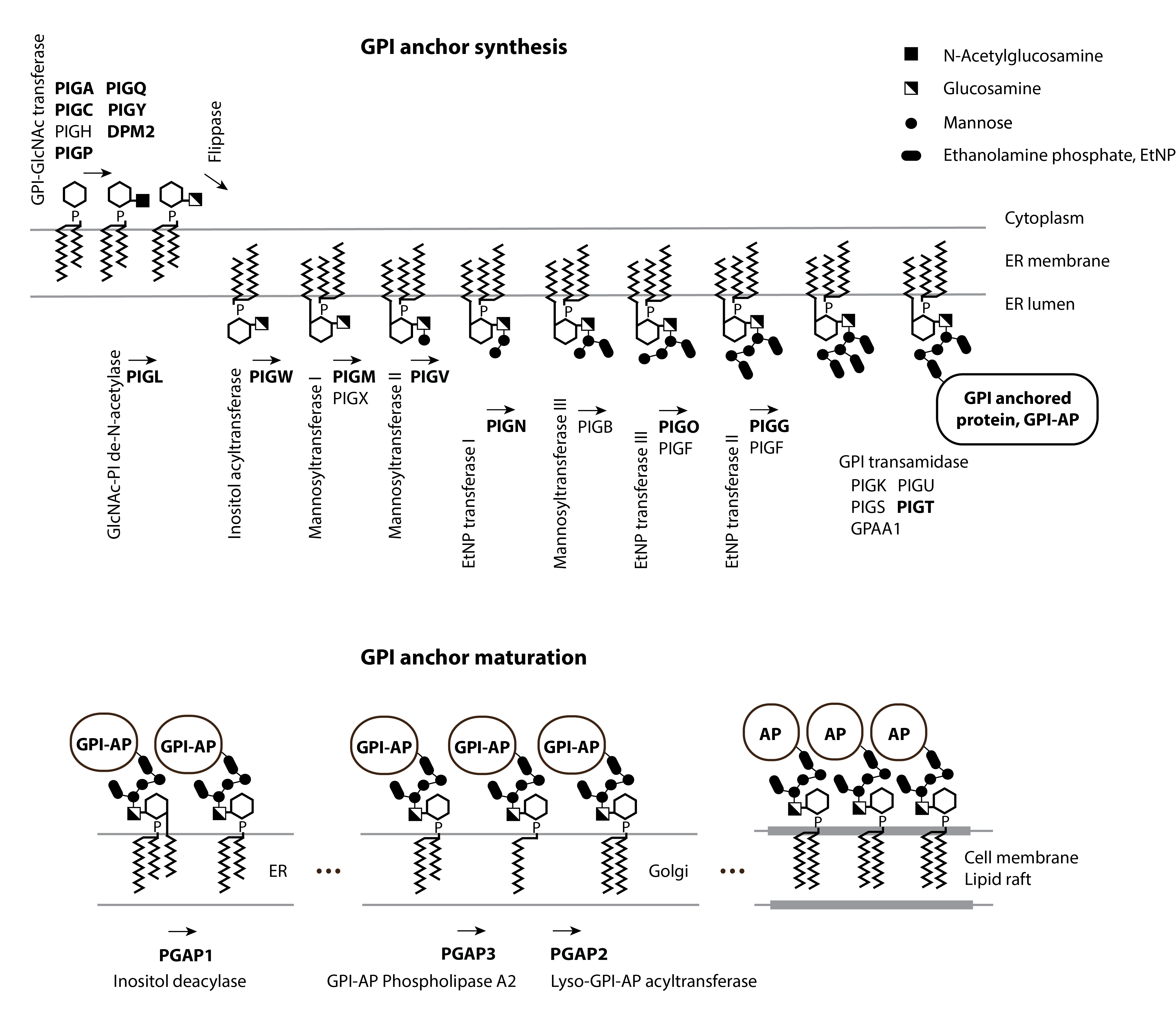


Figure S1: GPI anchor synthesis and maturation pathway. Genes with described pathogenic mutations are depicted in bold.

Figure S2: Mutational overview for *PIGA*, *PIGN, PIGT, PIGV*, and *PGAP3*. Mutations colored in red are described in this work for the first time. Pathogenic missense mutations have been observed for almost all coding exons. LoF variants have not been reported in homozygous state but have only been observed in compound heterozygotes. PGAP3 is exceptional in two respects: it is so far the only gene for which patients with homozygous LoF variants and pathogenic noncoding mutations have been described (Abdel 2017, Knaus2016).

Figure S3: The performance for pair-wise discrimination of gene-phenotypes was evaluated by the area under the receiver operating characteristic curve (AUC). The predictions achieve significantly better values then expected under a model of random chance (diagonal between true positive rate on the y-axis and true negative rate on the x-axis). It is notable that the PGAP3 cohort contains ten patients from Egypt (Abdel 2017). It could not be ruled out that ethnicity as a confounding factor had a positive effect on the discriminatory power between patients with defects in *PGAP3* and any other gene.
